# Epigenetic regulation of double c2 like domain beta (*Doc2b*) in cervical cancer

**DOI:** 10.1186/1755-8166-7-S1-I22

**Published:** 2014-01-21

**Authors:** K Satyamoorthy, Samatha Bhat, Harish Rotti, K Shamaprasada

**Affiliations:** 1 Manipal Life Sciences Center, Manipal University,Manipal, Karnataka, India

## 

Associations of genetic changes and aneuploidy with tumor growth are traditionally attributed to alterations in DNA sequence manifested as mutations, deletions and amplifications. Inactive tumor suppressor genes could serve as drivers of tumor progression due to not only altered or lack of protein function but may also contribute to phenotypic changes that may provide distinct growth advantage in a hostile environment in the host. Human variation is also due to epigenetic alterations and heritable change that leads to altered gene expression; the functional consequence of which may contribute to definitive trait. A number of key regulatory genes associated with epigenetic silencing due to DNA methylation in cervical cancer have been reported. Elucidation of differentially methylated genes may identify new targets and further strengthen our understanding of molecular mechanism governing pathogenesis of cervical cancer. Thus, to identify DNA methylation regulated genes in cervical cancer, we have employed DMH based microarray experiments in pre-malignant and malignant cervical sample. Microarray data analysis and validation using bisulfite genomic sequencing lead to the identification of several CpG island as altered during cervical carcinogenesis and showed the potential for early screening of cervical cancer. One of the candidate gene identified was Double C2 like Domain beta (DOC2B), a key calcium regulator protein whose alteration has never been linked to cancer. We provide evidence that DOC2B is depressed in cervical cancer due to promoter hypermethylation and act as a novel tumor suppressor gene by regulating multiple pathways in cervical cancer.

